# Low-Complexity Timing Correction Methods for Heart Rate Estimation Using Remote Photoplethysmography

**DOI:** 10.3390/s25020588

**Published:** 2025-01-20

**Authors:** Chun-Chi Chen, Song-Xian Lin, Hyundoo Jeong

**Affiliations:** 1 Electrical Engineering Department, National Chiayi University, Chiayi 600355, Taiwan; 2 Department of Biomedical and Robotics Engineering, Incheon National University, Incheon 22012, Republic of Korea

**Keywords:** remote photoplethysmography (rPPG), remote heart rate estimation, timing correction

## Abstract

With the rise of modern healthcare monitoring, heart rate (HR) estimation using remote photoplethysmography (rPPG) has gained attention for its non-contact, continuous tracking capabilities. However, most HR estimation methods rely on stable, fixed sampling intervals, while practical image capture often involves irregular frame rates and missing data, leading to inaccuracies in HR measurements. This study addresses these issues by introducing low-complexity timing correction methods, including linear, cubic, and filter interpolation, to improve HR estimation from rPPG signals under conditions of irregular sampling and data loss. Through a comparative analysis, this study offers insights into efficient timing correction techniques for enhancing HR estimation from rPPG, particularly suitable for edge-computing applications where low computational complexity is essential. Cubic interpolation can provide robust performance in reconstructing signals but requires higher computational resources, while linear and filter interpolation offer more efficient solutions. The proposed low-complexity timing correction methods improve the reliability of rPPG-based HR estimation, making it a more robust solution for real-world healthcare applications.

## 1. Introduction

With the rise of modern healthcare monitoring, heart rate has become a key indicator of cardiovascular health, physical fitness, and stress levels, making it essential for assessing overall well-being. However, traditional heart rate (HR) measurement methods require specialized equipment or in-person consultations, which can be inconvenient, especially during pandemic restrictions or in remote areas. For instance, although electrocardiography (ECG) is widely used to accurately estimate heart rate, it requires specialized equipment, such as electrodes, which makes it inconvenient for developing remote healthcare systems [1]. Photoplethysmography (PPG) provides an effective means of collecting physiological information in a non-invasive manner using small-sized sensors. However, its estimation capability is vulnerable to low-perfusion states [2], and it still requires contact with a sensor, which can increase the risk of transmission caused by viruses. Although ballistocardiograph (BCG) enables us to collect physiological data in a non-invasive and contactless manner, it is highly susceptible to noise from body movements or external vibrations [3,4]. To overcome these challenges, remote photoplethysmography (rPPG) offers a non-contact method that accurately estimates heart rate by analyzing subtle skin color changes captured in video images [5,6,7]. The rPPG signal, derived from these images, can detect subtle changes in microvascular blood volume caused by heartbeats, enabling non-invasive data collection [8,9,10]. Due to its non-invasive nature and compact design, rPPG supports telemonitoring via mobile devices and is increasingly used in various healthcare platforms [11,12].

To accurately estimate heart rate, various rPPG analysis algorithms have been developed to extract HR information, each with distinct strategies and benefits. Typical methods involve face tracking and skin segmentation to define regions of interest (ROIs), followed by signal processing and feature extraction for HR estimation [13,14,15]. Once the ROI is set, signal analysis techniques like independent component analysis (ICA) can be used to separate the pulse signal from noise by isolating independent components from the mixed RGB channels, thereby improving the accuracy of heart rate estimation [16]. Similarly, principal component analysis (PCA) can be employed to reduce the dimensionality of the data, simplifying the signal processing and lowering computational complexity while retaining key features essential for accurate HR estimation [17]. To address motion artifacts that often occur during rPPG measurement, model-based techniques can combine signal processing with physiological models to effectively separate the pulse signal from these disturbances [13]. Additionally, effective illumination normalization methods can improve ROI detection by maintaining consistent lighting conditions, thereby enhancing face tracking accuracy and reducing the impact of motion artifacts [18].

Recent rPPG research has applied deep learning (DL) to directly estimate HR from camera images, reducing the need for multiple processing stages and automating feature selection. Although DL-based algorithms require larger training datasets, they have the potential to achieve higher accuracy in HR estimation compared to traditional methods. For example, HR-CNN [19] uses convolutional neural networks (CNNs) to extract rPPG signals and estimate heart rate, while DeepPhys [20] employs convolutional attention networks with a motion-based model to improve accuracy under varied motion conditions. Beyond CNNs, long short-term memory (LSTM) networks can further enhance HR estimation by capturing temporal dependencies in time-series data, effectively reducing noise in rPPG signals [21,22]. Combining CNNs and LSTMs has also proven effective, as CNNs excel at detecting ROI in individual frames and LSTMs capture temporal patterns across frames, leading to more accurate heart rate estimation [23]. Transformers have recently emerged as a prominent focus in deep learning research, with applications in processing rPPG signals and HR estimation. Their exceptional ability to handle one-dimensional sequences, such as natural language and time-series data, has led to the development of specialized versions, like video transformers, which are designed to effectively process video frames and extract rPPG signals. For instance, the instantaneous transformer utilizes a spatial backbone based on DeepPhys and incorporates a temporal aggregation module to capture temporal correspondences within the signals [24]. PhysFormer employs a temporal difference transformer to enhance quasi-periodic rPPG features using temporal difference-guided global attention and further refines the local spatio-temporal representation to mitigate interference [25,26]. After the success of the transformer in rPPG signal estimation, several additional algorithms have been proposed, including Radiant [27], EfficientPhys [28], RhythmFormer [29], MaskFusionNet [30], and PhysMamba [31]. Furthermore, an open-source framework has been introduced, integrating advanced algorithms for rPPG signal estimation and providing benchmark datasets, serving as a valuable resource for advancing research for further research in this field [32].

However, these approaches generally assume consistent interframe durations without missing frames. In practice, video capture can often experience irregular sampling rates and frame drops, leading to inaccuracies in HR estimation. Furthermore, there is limited research focusing on timing correction for heart rate estimation under such conditions. This study introduces a timing correction method designed to address accuracy challenges in HR estimation using rPPG. The proposed low-complexity approach is ideal for lightweight devices that lack precise imaging equipment or the capacity for complex computations, enhancing the precision and reliability of HR estimation.

## 2. Materials and Methods

In practical applications, video capture for rPPG is often subject to irregular sampling intervals due to frame drops or inconsistent frame rates. Heart rate estimation is typically performed by detecting peaks in the frequency of the rPPG signal corresponding to individual heartbeats. These irregularities can lead to inaccuracies in heart rate estimation, as most HR estimation methods assume a consistent sampling rate. To address this issue, we present low-complexity time correction methods for HR estimation based on interpolation. Interpolation methods, including linear, cubic spline, and cascaded integrator–comb filter interpolation, offer computational complexity of O(n), where *n* represents the number of data samples [33,34,35]. These techniques can efficiently normalize the sampling intervals of the rPPG signal, ensuring reliable and accurate HR estimation.

### 2.1. Interpolation

**Figure 1:**
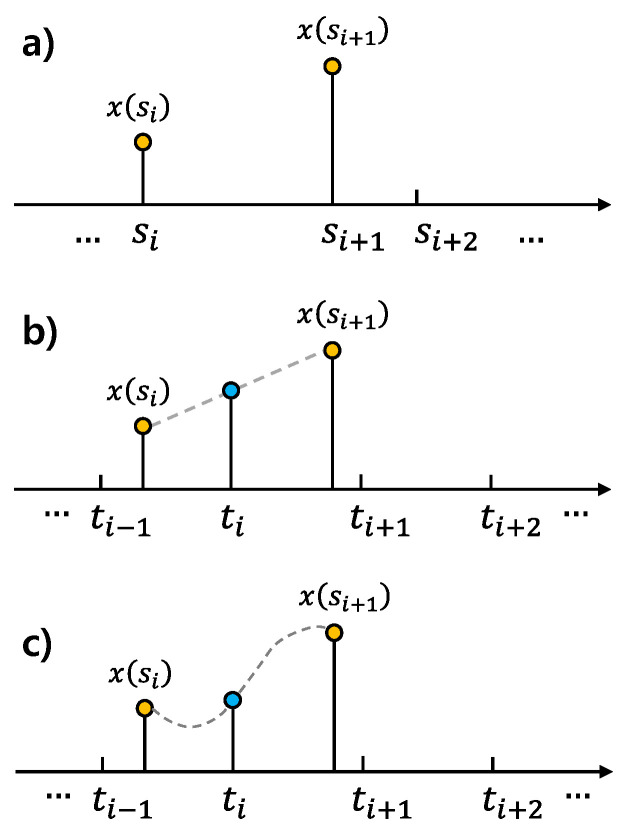
(**a**) Irregular timing samples. (**b**) Linear interpolation. (**c**) Cubic spline interpolation.

The HR estimation process involves capturing video frames using a camera and simultaneously logging the exact timestamps of each captured frame. To correct irregular sampling, we apply interpolation techniques, as illustrated in Figure 1, on the recorded rPPG signal based on the logged timestamps. Interpolation allows us to estimate signal values at uniformly spaced time intervals, ensuring that the rPPG signal is resampled at a consistent rate. Let $s_{1},s_{2},...,s_{n}$ represent the original, irregular timestamps, and let $x(s_{i})$ represent the rPPG signal values sampled at timestamp $s_{i}$. We define a uniformly spaced set of target timestamps $t_{1},t_{2},...,t_{n}$, where $t_{i}$ represents the $i^{th}$ uniformly spaced timestamp in the corrected time domain. The rPPG signal is then resampled to estimate the value $x(t_{i})$ corresponding to the target uniformly spaced timestamp.

Linear interpolation is a quick approximation used to interpolate values between two sampled data points by assuming a linear relationship between them. The interpolation formula is as follows:

(1) $$\begin{array}{c} x\left(t_{i}\right)=x\left(s_{i}\right)+\frac{x\left(s_{i+1}\right)-x\left(s_{i}\right)}{s_{i+1}-s_{i}}\times \left(t_{i}-s_{i}\right), \end{array}$$

where $s_{i}\leq t_{i}<s_{i+1}$, and $t_{i}$ is the target time point at which the resampled value $x(t_{i})$ is calculated. Unlike linear interpolation, which connects points with straight lines, cubic spline interpolation ensures that the curve is smooth at the sampled data points by using cubic polynomial curves. For each sample interval $\left(s_{i},s_{i+1}\right)$, the cubic spline is represented by a polynomial of the following form:

(2) $$\begin{array}{c} x_{i}\left(t_{i}\right)=a_{i}{\left(t_{i}-s_{i}\right)}^{3}+b_{i}{\left(t_{i}-s_{i}\right)}^{2}+c_{i}\left(t_{i}-s_{i}\right)+d_{i}, \end{array}$$

where $a_{i},b_{i},c_{i},d_{i}$ are the coefficients to be determined using boundary conditions, along with the continuity of the first and second derivatives at sample points. By maintaining derivative continuity, cubic spline interpolation provides a smooth and continuous curve through the sample points.

### 2.2. Filter Interpolation

**Figure 2:**
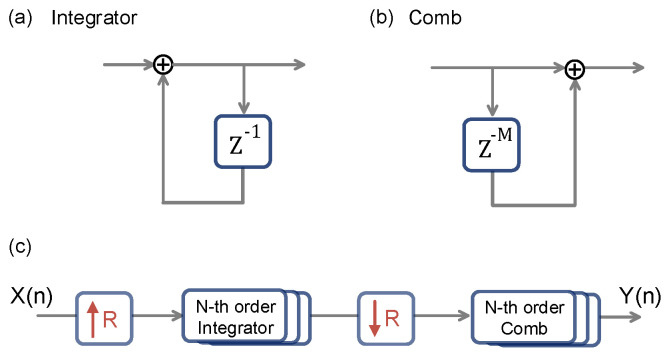
CIC filter. (**a**) The integrator filter accumulates the input signal by summing its current and previous values, effectively smoothing the signal and applying a low-pass filtering effect. (**b**) The comb filter functions as a differentiator by subtracting the delayed signal from the current signal, with the delay determined by parameter *M*. (**c**) CIC filter with up/down sampling rate R. The CIC filter structure consists of upsampling by a factor R, followed by cascaded N-order integrator filters. After downsampling, the signal is passed through N-order comb filters, effectively achieving filter interpolation.

Filter interpolation estimates values between discrete sample points by applying a filtering process. This approach is commonly used in signal processing to create smooth transitions between sampled points or to reconstruct a continuous signal from discrete samples. Furthermore, filter interpolation can reduce noise, minimize aliasing, and it generate a smoother and more coherent signal. For low-complexity applications, cascaded integrator–comb (CIC) filters are an efficient solution for interpolation. CIC filters are digital filters that do not require multipliers, making them highly suitable for hardware implementations due to their computational efficiency. As illustrated in Figure 2, the sampled data are first upsampled by inserting zeros between data points to increase the resolution. The upsampled signal is passed through a cascade of N-th order integrators, which smooth out the sharp transitions caused by the zero insertion. After integration, the signal is downsampled by a factor of *R* and then passed through a cascade of N-th order comb filters. These comb filters act as differentiators by subtracting signals delayed by *M*, thereby refining the signal and producing the resampled target signal. By combining decimation with a simple filter structure that requires no multipliers, CIC filters significantly reduce computational complexity. This makes CIC filters especially advantageous in interpolation applications that demand high data throughput with minimal computational overhead.

## 3. Results

In practical applications, signals are typically sampled at regular intervals. However, hardware limitations, such as insufficient processing power, limited memory capacity, or low-quality image sensors, can lead to irregular sampling times or missing data. For instance, devices with low processing power may struggle to process high-resolution image frames in real time, resulting in dropped or delayed frames. Similarly, limited buffer sizes or network bandwidth in streaming scenarios can cause frame loss or irregular intervals during data transmission. These inconsistencies can degrade the accuracy and reliability of signal processing tasks, ultimately affecting HR estimation. Though higher-quality sensors or hardware with enhanced processing power and memory can improve data reliability, environmental factors still introduce uncontrollable variables. Therefore, we adopt signal processing algorithms for timing correction to improve HR estimation under such conditions. To evaluate the impact of irregular sampling intervals and missing samples on signal reconstruction and HR estimation, we compare the performance of various reconstruction techniques, including linear interpolation, cubic spline interpolation, and filter interpolation. For CIC filter interpolation, the resample rate R is set to 10, a cascaded order of N=4, and a subtracting delay of M=2 in the comb filter. Although there are no official normal limits for resting heart rate, we focus on signals with a frequency bandwidth between 0.8 and 1.8 Hz, corresponding to a resting heart rate range of 50 to 100 beats per minute for normal adults [36,37]. The comparison emphasizes key metrics such as root mean squared error (RMSE) and heart rate estimation precision under varying conditions. These methods are applied to simulated datasets with different levels of irregularity and missing data to assess their effectiveness. As the number of missing samples increases, a corresponding rise in RMSE is observed, underscoring the degradation in signal fidelity. The cubic spline exhibits a more robust performance against increasing gaps in data.

### 3.1. Signal Interpolation Evaluation

**Figure 3:**
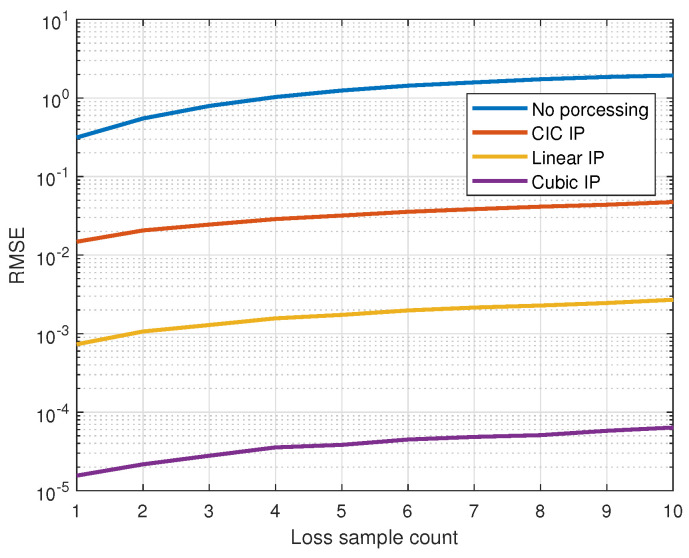
Root mean squared error (RMSE) versus the count of lost samples, comparing signal reconstruction accuracy with no processing and different interpolation methods. Each simulation was conducted for a specific loss sample count, comparing signals sampled under normal conditions with unprocessed signals and those reconstructed using interpolation methods.

**Figure 4:**
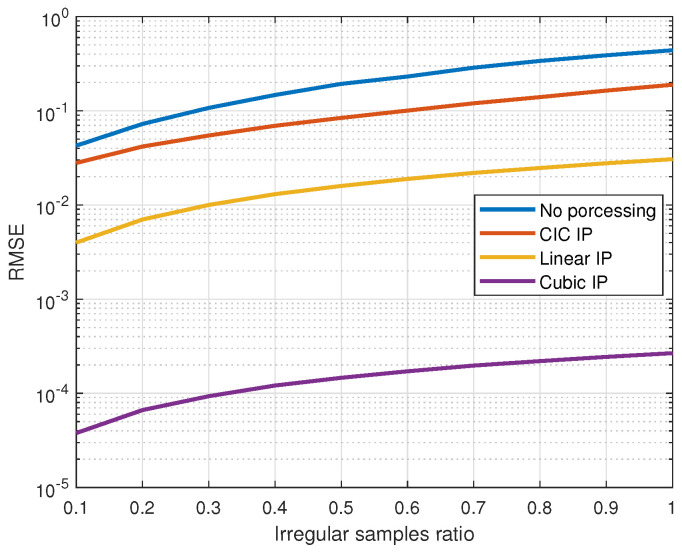
Root mean squared error (RMSE) versus the irregular sample ratio, comparing signal reconstruction accuracy with no processing and different interpolation methods. Each simulation was conducted for a specific irregular sample ratio, comparing signals sampled under normal conditions with unprocessed signals and those reconstructed using interpolation methods.

We assess the performance of various interpolation methods under conditions of missing samples and irregular sampling intervals. Each trial begins by generating signals at a sampling rate of 1 kHz, characterized by unit power and a random phase within the frequency bandwidth corresponding to the resting heart rate. Under normal sampling conditions, the signals are regularly sampled at a target rate of 25 Hz, resulting in a fixed time interval of 40 ms between samples. Otherwise, irregular timing samples occur when sampling bias introduces variations in these intervals. During each trial, the sampling times are recorded over a 30 s duration. The evaluation is conducted under two conditions: one in which samples are missing from the signal and another where a fixed proportion of the signal exhibits sampling bias. The irregular sampling bias is modeled as a normal random distribution, with a standard deviation equal to the target sampling period, and the maximum bias is constrained to the sampling period. To assess signal distortion in the frequency domain, we compare the effects of varying rates of irregular sampling and the counts of loss samples. Across 1000 trials, we compare signals sampled under the normal condition with those reconstructed under a specific non-ideal condition by applying interpolation methods for timing correction. RMSE is used to quantify the difference, reflecting how effectively interpolation restores signals under non-ideal conditions. Figure 3 illustrates the relationship between the RMSE and the count of loss samples, providing a quantitative analysis of how data loss affects the accuracy of the signal reconstruction across different interpolation methods. Without applying a timing correction method, even a small number of sample losses can result in an average error ranging from 0.3 to 1.1 of the signal amplitude. However, the proposed timing correction method significantly reduces distortion. For instance, even with 10 sample losses, the RMSE achieved using the proposed method is notably smaller compared to cases without timing correction. Figure 4 shows the relationship between the RMSE and the irregular sample ratio, highlighting the impact of non-uniform sampling on reconstruction accuracy. While the effects of irregular sampling are less severe than those of data loss, they still cause notable signal distortion. Severe signal distortion is likely to occur and alter the main components of the signal without any processing. The cubic spline interpolation exhibits a more robust performance against increasing losses and irregular samples in data, but it requires high computational effort to reconstruct smooth signals. In contrast, CIC filter interpolation has low computational complexity, with minor discrepancies depending on its resampling rate. It remains capable of restoring the signal to an adequately low error level.

### 3.2. Heart Rate Evaluation

**Figure 5:**
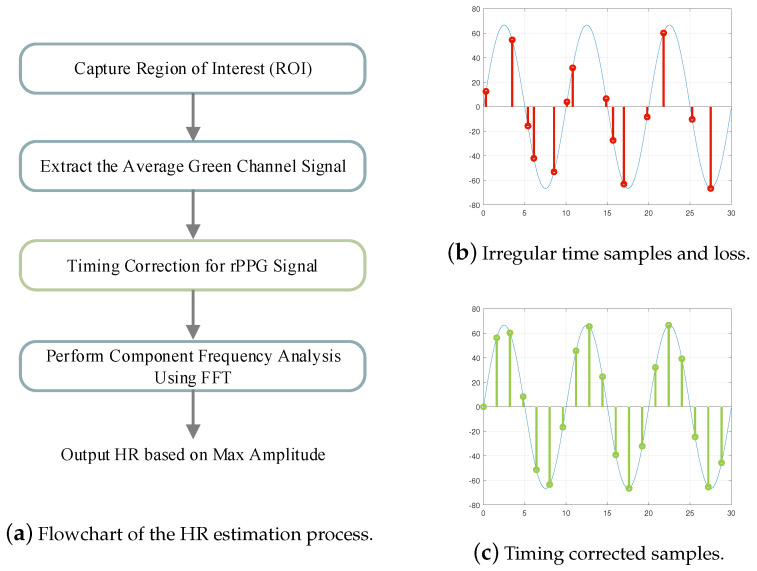
Timing correction for rPPG signal. (**a**) The flowchart illustrates the steps in HR estimation, including ROI extraction, green channel averaging, timing correction, frequency analysis using FFT, and HR determination based on the maximum amplitude. (**b**) The rPPG signal with irregular time samples and sample loss. (**c**) Timing correction improves HR estimation by interpolating rPPG samples to create a signal with consistent time intervals.

**Figure 6:**
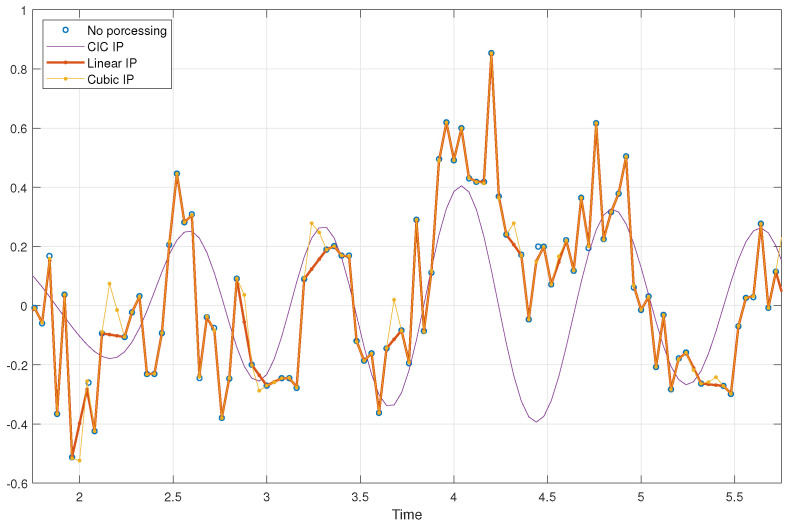
Comparison of the actual rPPG signal with sample loss alongside the interpolated signals obtained using linear, cubic spline, and CIC filter interpolation methods.

Accurate heart rate monitoring is crucial for numerous medical and fitness applications, as it provides key insights into cardiovascular health status. We evaluated the performance of different interpolation methods for HR estimation under irregular sampling intervals and missing data. The dataset consists of recordings from 22 adult subjects, primarily aged between 20 and 23, of Asian ethnicity, with a gender distribution of one-third female and two-thirds male. The measurements were conducted in an indoor setting at a temperature range of 26–27°C and a relative humidity of 45–55%. The camera primarily focused on capturing the subject’s face, maintaining a distance of less than 1 meter. Data were captured using a Realtek USB2.0 HD UVC WebCam (Realtek Semiconductor Corp., Hsinchu, Taiwan), operated via the Python (version 3.8.2) OpenCV module, with a nominal sampling rate of 25 frames per second (fps, $T_{s}=40$ ms) and a resolution of 640×480. Additional settings used for data capture are detailed in Table 1. The subjects were recorded at rest while maintaining stable heart rates, with timestamps logged for each frame. Once the measurement conditions stabilized, a 32 s recording was conducted for HR estimation. The actual sampling rate ranged between 20 and 30 fps, exhibiting an irregular sampling bias with a mean of $0.03T_{s}$ and a standard deviation $0.13T_{s}$. Additionally, two subjects experienced sample loss, accounting for approximately 1.2% of the recording samples. The recording parameters are summarized in Table 2. Although the experiments were limited to a small group of individuals with similar ages and skin tones, the primary objective of this study was to evaluate the effectiveness of timing correction in enhancing HR estimation. Each subject wore a wrist-based blood pressure monitor to provide heart rate measurements, which served as a reference for evaluating the impact of timing correction under timing-related issues. Figure 5 illustrates the steps involved in the heart rate (HR) estimation process using rPPG. The rPPG signal is primarily derived from the facial region between the eyes and mouth, where heart rate predictability is most prominent [38]. Furthermore, the average of the green channel is used for HR estimation, as it provides the most reliable rPPG information related to blood volume changes compared to other channels [6]. Timing correction is applied to address irregular time sampling and sample loss, ensuring consistent time intervals for accurate analysis, as illustrated in Figure 5b,c. FFT is then used to analyze the component frequency of the corrected rPPG signal, and the heart rate in beats per minute is determined by identifying the dominant frequency of the rPPG signal. Figure 6 illustrates the actual rPPG signal with sample loss alongside the interpolated signals obtained using linear, cubic spline, and CIC filter interpolation methods. Linear interpolation connects sample points with straight lines, while cubic spline interpolation creates smooth connections using cubic polynomial curves. CIC filter interpolation reconstructs signals by applying low-pass filters to reduce high-frequency noise and smooth the signal.

**Figure 7:**
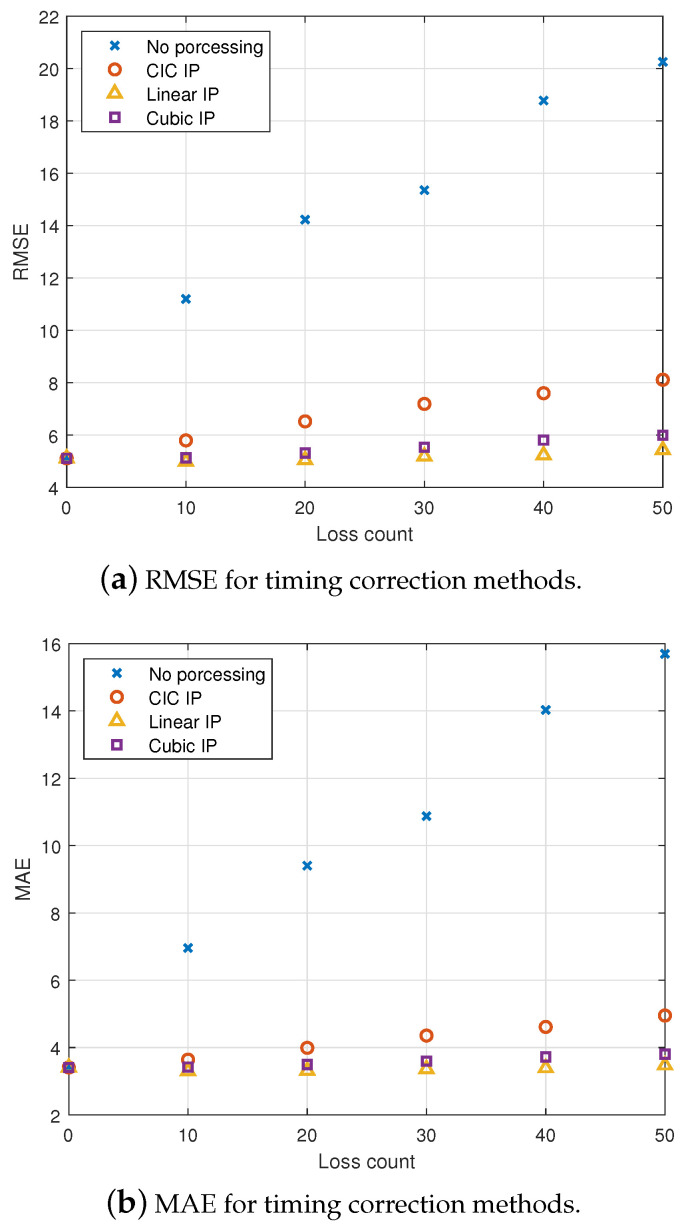
Comparison of interpolation methods for rPPG signals. (**a**) RMSE and (**b**) MAE metrics for various interpolation methods applied to rPPG signals, demonstrating the comparative accuracy of each approach in HR estimation.

To evaluate the impact of irregular timing samples and sample loss on HR estimation, we conducted the following simulations: For each simulation, specific numbers of samples were randomly dropped from the actual rPPG signal, and a 30 s rPPG signal was analyzed to test HR estimation using different timing correction methods. To assess performance, RMSE and MAE were calculated for each simulation. RMSE, due to its quadratic nature, emphasizes larger errors and is sensitive to outliers, while MAE provides the average of absolute errors, offering a straightforward measure of overall error magnitude when outliers are less influential. A total of 1000 simulations were performed, and the final averaged results are summarized in Table 3. Figure 7 presents the RMSE and the mean absolute error (MAE) metric for different interpolation methods applied to the rPPG signals. As HR estimation is based on identifying the dominant frequency with maximum power, it exhibits a certain level of resistance to sample loss and irregular timing samples. The instability of the sampling frame rate has little impact on the determination of heart rate from the dominant frequency, as accuracy can be maintained even without the use of interpolation methods. However, sampling loss can lead to errors in heart rate estimation without interpolation correction. By applying interpolation methods, a certain level of accuracy in heart rate estimation can be maintained, even with significant sampling loss. The estimated signal remains stable without introducing sample loss in the simulation, leading to consistent HR estimation without variation. Linear and cubic spline interpolation demonstrate better signal reconstruction, resulting in more accurate HR estimation even under conditions with significant sample loss. CIC filter interpolation exhibits slightly higher reconstruction errors and variation as sample loss increases. For edge computing applications requiring extremely low computational complexity, linear and CIC interpolation can provide efficient interpolations. Additionally, if low-pass filtering is required for further processing of the rPPG signal, CIC filter interpolation serves as a suitable solution for achieving reliable HR estimation.

## 4. Discussion

This study focuses on timing correction as a front-end processing step for HR measurement using rPPG. Effective timing correction methods enable accurate reconstruction of rPPG signals and HR estimation, even under conditions of irregular sampling and sample loss. The proposed methods are designed with low computational complexity, making them suitable for implementing various healthcare services using rPPG on mobile and edge computing devices.

We analyze the effect of timing correction on the RMSE and MAE of HR estimation, as heart rate monitoring is an increasingly important application of rPPG in remote healthcare services and wearable technologies. This study investigates the effects of frame loss and timing correction on the accuracy of heart rate estimation using rPPG signals, filling a gap in the existing literature. Direct comparisons with previous studies are challenging due to the lack of research on timing correction algorithms for rPPG signals. However, benchmarking datasets show that state-of-the-art algorithms achieve RMSE and MAE values of less than 10 for HR estimation [39,40,41]. Similarly, studies like Ze Yang et al. [42] report an MAE of less than 9 and an RMSE of less than 13 under low-illumination conditions, which can have a similar effect to frame loss. Based on the results of related studies, cutting-edge algorithms achieve MAE and RMSE values for heart rate estimation of less than 5 and 10, respectively. Our experimental results show that the RMSE and MAE gradually increase with frame loss, which can exceed acceptable limits when frame loss exceeds 10 frames in practical conditions. However, the proposed timing correction method can reduce RMSE to less than 8, ensuring practical usability. Moreover, without timing correction, MAE exhibits an approximately linear increase with frame loss, while the proposed algorithm minimizes its sensitivity to frame loss. These RMSE and MAE values demonstrate the importance of the timing correction method for accurately estimating heart rate using rPPG signals in practical applications.

To further expand the scope of this research and enhance the applicability of these timing correction methods across diverse rPPG applications, this study discusses current limitations and explores potential future research directions. First, incorporating a more diverse population with varying skin tones and age groups would provide valuable insights into the robustness of the proposed methods. For instance, rPPG signals captured from individuals with darker skin tones often show higher accuracy because darker skin makes blood vessels more visible [43]. However, this study primarily collected data from Asian participants with similar skin tones, limiting the evaluation of how skin color affects the effectiveness of timing correction. Expanding the dataset to include individuals with diverse dermatological characteristics would facilitate a more comprehensive assessment of timing correction methods and their adaptability across broader populations.

Additionally, the applicability of the proposed methods can be enhanced by assessing their performance in real-world conditions involving motion artifacts and varying light conditions. Although rPPG provides a non-invasive solution for HR estimation, it remains vulnerable to interference from these factors. Investigating the interaction between timing correction, motion artifacts, and reconstructed signals under such conditions is essential for improving robustness. Furthermore, incorporating auxiliary equipment like near-infrared cameras could enhance rPPG accuracy in low-light environments [44,45]. Future studies could evaluate the effectiveness of timing correction on datasets collected under these challenging conditions, providing insights into its adaptability to diverse and complex scenarios. Lastly, the impact of timing correction on estimating additional physiological parameters, such as blood pressure and blood oxygen saturation ($SPO_{2}$), could be explored [46,47,48]. These additional studies could further enhance the versatility of the rPPG technique and the proposed timing correction method.

## 5. Conclusions

This study evaluates heart rate (HR) estimation under irregular sampling intervals and missing data, both of which can distort rPPG signals. While frame rate variations have a minor impact on detecting the dominant frequency of the rPPG signal, missing samples can shift the main frequency and cause significant errors without interpolation, leading to inaccurate HR estimates. Without timing correction, RMSE increases rapidly from 5.1 to 19.5, and MAE rises from 3.4 to 15.1 as sample loss increases. However, applying timing correction using interpolation effectively controls RMSE within 7.1 and MAE within 4.5, maintaining HR accuracy even under significant sample loss. While cubic spline interpolation provides robust performance in reconstructing signals with gaps and irregularities, its high computational demand may limit its practical use in real-time applications. In contrast, linear and CIC interpolation offer efficient, low-complexity solutions. CIC filter interpolation, in particular, offers a reliable solution when low-pass filtering of rPPG signals is needed for further processing. Overall, applying interpolation methods enhances reliable HR monitoring, particularly in situations with inconsistent data capture. Choosing the appropriate interpolation technique can effectively balance accuracy and efficiency, offering valuable insights for improving HR estimation from rPPG signals.

## Figures and Tables

**Table 1:** Detailed camera settings used for data capture.

Parameter	Value
Brightness	55
Saturation	64
Contrast	50
Hue	0
Exposure	−6 (EV)
Gamma	300

**Table 2:** Summary of the parameters for the experimental recordings, including the number of subjects, sampling rates, recording period, and total number of recorded samples.

Parameter	Value
Subjects	22
Actual sampling rate	20–30 fps
Nominal sampling rate	25 fps
Recording duration	32 s
Recording samples	800
FFT samples	750

**Table 3:** Comparison of RMSE and MAE results for different timing correction methods in HR estimation under specific sample loss conditions.

RMSE (Mean/Standard Deviation)				
**Loss Count**	**No Processing**	**CIC IP**	**Linear IP**	**Cubic IP**
0	5.11/0.00	5.11/0.00	5.11/0.00	5.11/0.00
10	11.20/1.35	5.80/1.42	4.99/0.25	5.13/0.38
20	14.23/1.47	6.52/1.74	5.05/0.50	5.32/0.74
30	15.36/1.37	7.19/1.89	5.19/0.78	5.54/0.97
40	18.78/1.36	7.60/1.97	5.24/0.83	5.81/1.21
50	20.25/1.20	8.11/2.11	5.44/1.06	6.00/1.31
**MAE (Mean/Standard Deviation)**				
**Loss Count**	**No Processing**	**CIC IP**	**Linear IP**	**Cubic IP**
0	3.41/0.00	3.41/0.00	3.41/0.00	3.41/0.00
10	6.96/0.84	3.64/0.64	3.30/0.22	3.42/0.24
20	9.40/1.17	3.99/0.84	3.32/0.28	3.50/0.39
30	10.88/1.10	4.36/0.94	3.36/0.36	3.60/0.48
40	14.03/1.18	4.61/1.05	3.39/0.38	3.72/0.59
50	15.69/1.22	4.95/1.17	3.48/0.49	3.80/0.63

## Data Availability

The data utilized in this study are available at https://github.com/aky3100/HR-Estimation (accessed on 15 January 2025).

## Abbreviations

The following abbreviations are used in this manuscript:

BCG	Ballistocardiograph
CIC	Cascaded integrator–comb
CNN	Convolutional neural network
DL	Deep learning
ECG	Electrocardiography
HR	Heart rate
ICA	Independent component analysis
LSTM	Long short-term memory
PCA	Principal component analysis
PPG	Photoplethysmography
ROI	Regions of interest
rPPG	Remote photoplethysmography
